# Synchronizing eye tracking and optical motion capture: How to bring them together

**DOI:** 10.16910/jemr.11.2.5

**Published:** 2018-05-07

**Authors:** Birgitta Burger, Anna Puupponen, Tommi Jantunen

**Affiliations:** University of Jyväskylä, Finland

**Keywords:** Eye movement, eye tracking, new media, intermodal processing, motion capture, technology, synchronization, methodology

## Abstract

Both eye tracking and motion capture technologies are nowadays frequently used in human sciences, although both technologies are usually used separately. However, measuring both eye and body movements simultaneously would offer great potential for investigating crossmodal interaction in human (e.g. music and language-related) behavior. Here we combined an Ergoneers Dikablis head mounted eye tracker with a Qualisys Oqus optical motion capture system. In order to synchronize the recordings of both devices, we developed a generalizable solution that does not rely on any (cost-intensive) ready-made / company-provided synchronization solution. At the beginning of each recording, the participant nods quickly while fixing on a target while keeping the eyes open - a motion yielding a sharp vertical displacement in both mocap and eye data. This displacement can be reliably detected with a peak-picking algorithm and used for accurately aligning the mocap and eye data. This method produces accurate synchronization results in the case of clean data and therefore provides an attractive alternative to costly plug-ins, as well as a solution in case ready-made synchronization solutions are unavailable.

## Introduction

Both eye tracking and motion capture technologies are nowadays widely used in human sciences (e.g., music research or sign language linguistics), although both technologies are usually used separately. However, the combination of measuring both eye and body movements simultaneously would offer great potential for investigating action-perception links and cross-modal interaction in human behavior in general, and in musical behavior and sign language in particular. Especially in communicative and joint actions, such as making music or dancing together, combining different data acquisition tools like motion capture and eye tracking would provide new and innovative possibilities for conducting research.

Possible research questions of interest could include whether performers in a musical ensemble coordinate eye and body movements to create successful joint performances or whether gaze directions reflect participants’ movements and interactive behaviors when dancing with another person. In the field of sign language research – in which eye behavior, together with the activity of the hands and other parts of the body, has been argued to be an important means to organize linguistic structure – possible research questions could include how exactly signers coordinate eye gaze and eye movements with manually produced linguistic units, and how the temporal alignment of eye and hand behaviors differ, for example, between native signers and sign language learners.

However, the biggest challenge in combining separate data acquisition technologies, such as motion capture and eye tracking, is reliably synchronizing the devices so that the data can either be recorded at the same time or be precisely aligned afterwards. Accurate synchronization of the different data streams is crucial for time-critical analysis of the data and for relating the different data streams to each other in order to answer the research questions at hand.

### Research using motion capture and eye tracking

Both technologies have been used separately in various research areas such as psychology, biomechanics, education, sports, linguistics, and music. Since the authors are mainly familiar with research in music and sign language, the following literature review will focus on these research fields.

#### Music and motion capture

In the music field, motion capture has been used to study gestures during music performance or spontaneous movement responses to music for example. In terms of performers’ gestures, work by, for instance, Thompson and Luck ( 32 )investigated expressivity during piano performances, finding increased movement in structurally important parts when playing expressively compared to playing without expression. Van Zijl and Luck ( 37 ) addressed the role of experienced emotions on movement characteristics during music performance, finding increased movement when playing with a sad expression compared to playing while being in a sad feeling. Glowinski et al. ( 12 ) studied the movements of a string quartet during performance, obtaining different head movement patterns in joint versus solo performances.

In music-induced movement, Burger, Thompson, Luck, Saarikallio, and Toiviainen ( 3 ) explored relationships between spontaneous full body movement and musical characteristics such as pulse clarity and spectral content, finding that clearer pulses and stronger spectral content in low and high frequencies encouraged participants to move more. Van Dyck et al. ( 36 ) showed that participants’ spontaneous movements increased with the presence of the bass drum. Carlson, Burger, London, Thompson, and Toiviainen ( 6 ) focused on personality characteristics in relation to music-induced movement, finding that participants with higher conscientiousness and lower extraversion show greater responsiveness to tempo changes. Haugen ( 15 ) studied music-dance relationships both in Brazilian Samba and Norwegian Telespringar, while Naveda and Leman ( 26 )investigated spatiotemporal representations in dance gestures of Samba and the Charleston.

Movement has also been studied from the perspective of perception. Vuoskoski, Thompson, Clarke, and Spence ( 38 ) showed stick-figure animations to participants and studied the perception of expressivity in musical performances, showing that the influence of the visual component seems stronger in the communication of expressivity compared to the auditory. Burger, Thompson, Saarikallio, Luck, and Toiviainen ( 4 ) investigated the attribution of emotions to music-induced movement by showing participants stick-figure animations of spontaneous dance movement, showing that dance was perceived rather as positive than negative emotions. Su and Keller ( 31 ) studied synchronization when perceiving stick-figure videos of dance movements of oneself and others, finding that participants, especially musicians, synchronized more accurately with others than with their own movements.

#### Sign language linguistics and motion capture

In sign language linguistics, motion capture has been used in a few works to investigate various linguistically relevant phenomena from an articulatory perspective. Concerning early work, Wilbur ( 41 )showed that there is a link between stressed sign production and certain kinematic variables such as displacement, velocity, and acceleration. Wilcox ( 42 ) , in turn, looked at the production of consecutive hand alphabets (i.e. fingerspelling) and showed, for instance, that the velocity peaks of the finger movements to target alphabets are a significant feature in the organization of fingerspelling.

More recently, Tyrone and Mauk ( 35 ) examined sign lowering (i.e. producing the sign lower than its citation form) in American Sign Language and found that it is affected in predictable ways by production rate, phonetic context, and position within an utterance (see also Mauk & Tyrone, 23 ). Jantunen ( 18 ), in turn, investigated whether the signed syllable – a sequential movement of the articulator(s) – could be empirically defined with the help of a single acceleration peak. He found that this was not the case, as the number of acceleration peaks in syllables could vary from zero to three and acceleration peaks could also be found outside the syllable domain. In another study, Jantunen ( 19 ) compared sign strokes ("signs") with non-strokes ("transitions") and established that there is a kinematic difference between them.

In a more recent work, Puupponen, Wainio, Burger, and Jantunen ( 29 ) analyzed the kinematic characteristics and functional properties of different head movements in Finnish Sign Language and showed that there is no perfect correspondence between their forms and functions, unlike results reported in some earlier studies.

#### Music and eye tracking

Eye tracking has been frequently used to study music (sight-) reading. When looking at amateur musicians Penttinen, Huovinen, and Ylitalo ( 27 ) found that more experienced musicians used shorter fixation times and more linear scanning of the notated music. Focusing on adult music students, Penttinen, Huovinen, and Ylitalo ( 28 ) found that performance majors showed shorter fixation durations and larger eye-hand spans. Professional performers had more efficient fixations that helped them anticipate difficulties and potential problems compared to non-musicians ( 27 ).

Hadley, Sturt, Eerola, and Pickering ( 14 ) found that harmonically incongruent melodies caused rapid disruption in eye movements and pupil dilation. Gruhn et al. ( 13 ) investigated differences between saccadic eye movements in musicians and non-musicians, finding that musicians had more express saccades, stronger voluntary eye control, and more stability in their fixations than non-musicians.

Laeng, Eidet, Sulutvedt, and Panksepp ( 21 ) found relationships between pupil dilation and musical chills, in that the pupil size increased around the moment of experiencing the chill. Gingras, Marin, Puig-Waldmüller, and Fitch ( 11 ) could predict pupillary responses from musicinduced arousal and individual differences – pupils dilated more for arousing or tense excerpts, in particular when the excerpts were liked less.

Fink, Geng, Hurley, and Janata ( 10 ) investigated the role of attention during music listening on pupil dilation, finding pupil dilations in deviants of complex musical rhythms. Woolhouse and Lai ( 43 ) studied participants’ eye movements while observing dance movements, finding more fixations on the upper rather than the lower body, as well as greater dwell times for the head than for torso, legs, or feet.

#### Sign language linguistics and eye tracking

In sign language linguistics, the use of eye tracking has been very rare. Concerning perception studies, Muir and Richardson ( 25 ) found that native signers tend to fixate on the upper face of the addressee, especially if the addressee is close by. Emmorey, Thompson, and Colvin ( 9 ) showed that this tends not to be the case for signing beginners who prefer to look at the mouth area. Wehrmeyer ( 39 ) showed that the viewing habits of deaf and hearing adults are also different in other contexts, for example, in watching sign language interpreted news broadcasts.

Concerning production studies, Thompson, Emmorey, and Kluender ( 33 ) found that signers’ gaze behavior is different depending of the type of the verb sign and how it is modified in the signing space. In a follow up study ( 34 ), they also showed that this gaze behavior is affected by signing skill. A recent study by Hosemann ( 17 ), however, suggested that the pattern found by Thompson et al. ( 33 ) may not be so systematic.

#### Combining motion capture and eye tracking

Within the music field, there have only been very few studies so far that tried to combine motion capture and eye tracking, while in sign language research, motion capture and eye tracking have not been used together before. In music-related research, Bishop and Goebl ( 1 ) study visual attention during duet performances, expecting that visual attention declines with repetition of the piece due to getting to know each other’s intentions. Marandola ( 22 ) investigated hand-eye synchronization in xylophone performance, suggesting that western musicians prepare for the hits to be performed with their gaze, while Cameroonian musicians tend to look away from the instrument.

### What is motion capture?

Different systems for recording motion capture are available ( 2 ). Inertial systems track the acceleration and orientation of sensors attached to participants/objects in three dimensions, while magnetic systems measure the three-dimensional position and orientation of objects in a magnetic field. Of more importance for this paper are camera-based systems, in particular infraredbased optical motion capture systems. In such systems, cameras send out infrared light that is reflected by (passive, wireless) markers attached to participants and/or objects, so that these reflections can be recorded by the cameras. These systems are composed of an array of several cameras chained in a row to represent the data in a three-dimensional space. Using a method called direct linear transformation, the system acquires the exact position and orientation of each camera, with respect to the others and the floor, to be able to create the three-dimensional representation of the capture space and triangulate the marker positions ( 30 ).

Since optical motion capture systems work with reflections (i.e., passive reflective markers) only, these markers need to be labeled to identify which body part or object each marker represents. Two main approaches for data labeling exist. Some systems, such as the ones manufactured by Vicon or OptiTrak, let the user define the locations of the markers and create a body model prior to the recording that is applied during the recording or post-processing. If the model works correctly, the data is labelled automatically. However, if the model fails (due to, for instance, marker loss), manual labeling is required. In the Qualisys system, the user first records the raw markers without any body model. Afterwards, one recording is labelled manually, from which a body model is created that is then applied to the other recordings to label them automatically. Also here, manual labeling is required, if the model fails. However, the model can be improved by updating it after each recording. The main challenge of optical systems is that occlusions of markers during the recording causes marker loss and gaps in the data. Thus, such occlusions should be prevented by careful marker placement and camera positioning before and during the recording.

Optical motion capture systems have high temporal and spatial resolutions, as recent systems track up to 10,000 frames per second and have a resolution of less than one millimeter. Normally in music- and sign language-related applications, standard capture speeds range from 60 to 240 Hz (most often 100-120 Hz), which is sufficient for capturing most relevant activities, such as playing instruments or dancing ( 2 ).

### What is eye tracking?

In the case of eye tracking, camera-based trackers are most widely used nowadays, with an infrared light source detecting the pupil by using the so called corneal reflections, resulting in a variety of different measures including the position or dilation of the pupil ( 16) ). Screen-based or stationary eye trackers are attached to the object to be tracked, usually a screen, with the participant placed in a stationary position in front of the screen and the tracking system. Mobile eye trackers, on the other hand, are head-mounted eye trackers worn like glasses so the participant can move in space while the tracker captures the eye movement and the scene being observed. Therefore, mobile eye trackers have two kinds of cameras, one (infrared-based) to record the eye/pupil and the other (regular pixel-based, fish-eye lensed) for the field or the scene, representing what the participant sees.

Eye trackers also require calibration, usually by providing four fixed points in space that the participant is asked to focus on one after another while keeping the head still (i.e., by only moving the pupils). With these four points, the system is able to combine the eye positions with the field video and display the focus of the gaze as a cross hair in the field video. Most mobile eye trackers track at rates of 50 or 60 Hz. Both mocap and eye tracking systems result in numerical data representations of the body and eye movement respectively that can be processed computationally.

### Synchronization of motion capture and eye tracking

Reliable and accurate synchronization between the motion capture system and eye tracker is crucial for relating both data streams to each other and time-critically analyzing the data. Different attempts have been developed. The two studies mentioned above have employed different methods. One possibility is to use (i.e., purchase) solutions offered by the manufacturers (e.g., using sync boxes or plug-ins like Bishop & Goebl, 1 )or alternatively use (analog) claps like Marandola ( 22 ) equipped with mocap markers recorded by the eye tracking glasses’ field camera. However, manual claps would require the researcher to manually synchronize the data, which is a rather time-consuming effort. Moreover, since the video (of the eye tracker field camera) recording the clap is based on (changing) pixels, the possibility of finding the exact frame to which the mocap data should be synchronized might be more challenging compared to working with digital representations of time series motion capture and eye movement data. Another potential challenge for synchronization might arise from differences in the starting times of the recordings of both eye tracking cameras. This would mean that the delay between the start of the eye camera and the field camera has to be additionally quantified for each recording, resulting in possible inconsistencies.

Ready-made solutions offered by the manufacturers are available for several motion capture system and eye tracker combinations, although not for all available eye systems. Furthermore, such a plug-in is relatively cost-intensive and usually requires a complicated technical setup using two computers (one for running the motion capture recording software, the other for running the eye tracker software – at least in case of the Qualisys motion capture system) that are linked via a wireless network connection, which might cause computer/system security issues or delays/lags in the processing. Other solutions (e.g., from Natural Point OptiTrack) work via a sync box connecting the different devices, for instance via a TTL signal and/or STPTE timecode (see below), which is also cost-intensive, possibly requiring engineering knowledge as cables might need customized connectors to fit into the available in- and outputs of the devices and computers.

Synchronizing different devices is a technically challenging problem. It is not only a challenge to ensure that recordings start at the same time, but also that they would not drift apart in time from each other during the recording (so one recording would be longer or have less frames recorded than the other). Another possibility could be that the sampling points of the different systems are locally misaligned (due to an unstable sampling rate) which is referred to as jitter. While high quality motion capture systems, such as the Qualisys system used in this case, exhibit close to zero drift and jitter (being one part per million according to the Qualisys costumer support), eye trackers are said to exhibit some drift and jitter ( 16 ).

Different ways to synchronize different devices have been developed and are used in industrial and research applications. One way is to send TTL (Transistor–transistor logic) triggers indicating the start and stop of a recording. Other developments include timecode and genlock/sync ( 24 ). Timecode, such as the SMPTE timecode, developed by the Society of Motion Picture and Television Engineers, is a standard in the film industry to link cameras or video and audio material. The SMPTE timecode indexes each recorded frame (or every second, third, etc. depending on the frame rate of the devices) with a time stamp, to offer synch points for post processing. However, such time codes can still cause jitter as well as drift if they are not strictly kept together by, for instance, using a central clock or a reference signal genlocking the devices. However, such devices are relatively expensive and require some engineering knowledge to set up correctly. Often, they also require a cable connection between the device and the recording computer. With this being less of a problem for the motion capture system (since the pulses would only be sent to the cameras), the (wireless) eye tracker would lose its mobility. Some systems offer wireless synchronization via WLAN, however, this is likely to introduce delays, inconsistencies, and data loss due to unreliability and loss of the signal.

Another option that has been developed to synchronize different devices is the lab streaming layer (LSL). The LSL is a system for the synchronized collection of various time series data over network. However, it requires programing and computer knowledge, especially if the motion capture and eye tracker systems at hand are not among the already supported devices. Thus, it might not be suitable and easy to use for everyone.

### Aim of this paper

In order to overcome such device-specific, hard- and /or software-based solutions, we aimed for a device-free, behavior-based approach to reliably synchronize the two systems that can be used with any combination of motion capture and eye tracking systems. This approach should be easy to perform for the participant and automatically processable by a computational algorithm to avoid manual synchronization of each separate recording. Such a solution has low demands on technical knowledge and could be used with any combination of eye tracker and motion capture system at no extra cost. Furthermore, the synchronization would be purely based on the numerical representations of both mocap and eye tracker data, so possible differences in recording beginnings of the different (eye tracker) cameras would not affect the synchronization accuracy.

This computational synchronization solution was developed in a pilot phase, and a refined version of it was subsequently tested in a second, larger data collection. This paper describes this development as well as the evaluation of the accuracy in comparison to manual synchronization of the recordings.

## Pilot phase – Methods and results

In order to develop a computational method to synchronize motion capture and eye tracker data, pilot data from six participants within a sign language experiment were collected. Data were simultaneously recorded using the motion capture system, the eye tracker, and an external (regular) video camera. An actual experiment setting was chosen so that we were able to collect the data in an authentic scientific scenario. During the experiment, each participant signed five different short stories, resulting in five recordings per participant.

### Equipment

We used a Qualisys Oqus 5+ infrared optical motion capture system (8 cameras mounted to the ceiling of the room) tracking at a frame rate of 120 Hz as well as an Ergoneers Dikablis Essential head mounted eye tracker (glasses) tracking at a frequency of 50 Hz (both eye and field camera). 120 Hz is the usual frequency at which we track motion capture data, being sufficient for whole body movement ( 2 ). 50 Hz is the only frequency at which this eye tracker operates.

### Procedure

**Figure 1.**
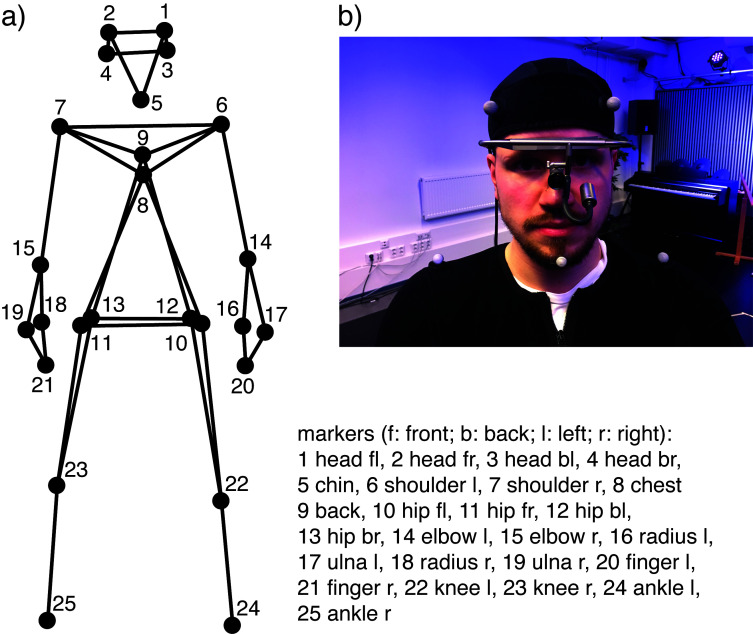
a) Mocap markers as a schematic representation and b) mocap markers and eye tracker attached to participant.

At the beginning of the recording session, participants were equipped with motion capture markers (25 in this case) and the eye tracker glasses, which were calibrated to the participant’s left eye (see Fig. 1).

**Figure 2.**
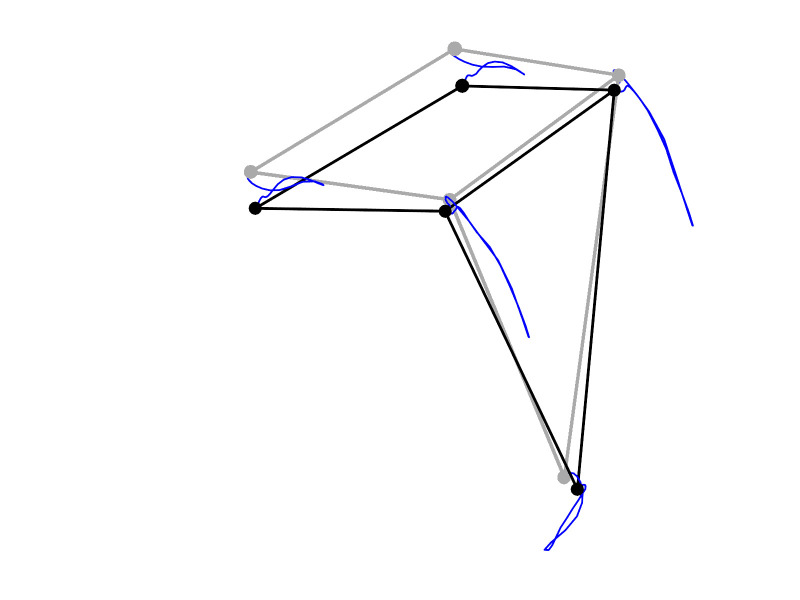
Trace illustration of the nod of the four head and the chin markers. The gray stick representation depicts the starting point, the black stick representation the end position, and the blue lines display the motion trajectory of the nod.

**Figure 3.**
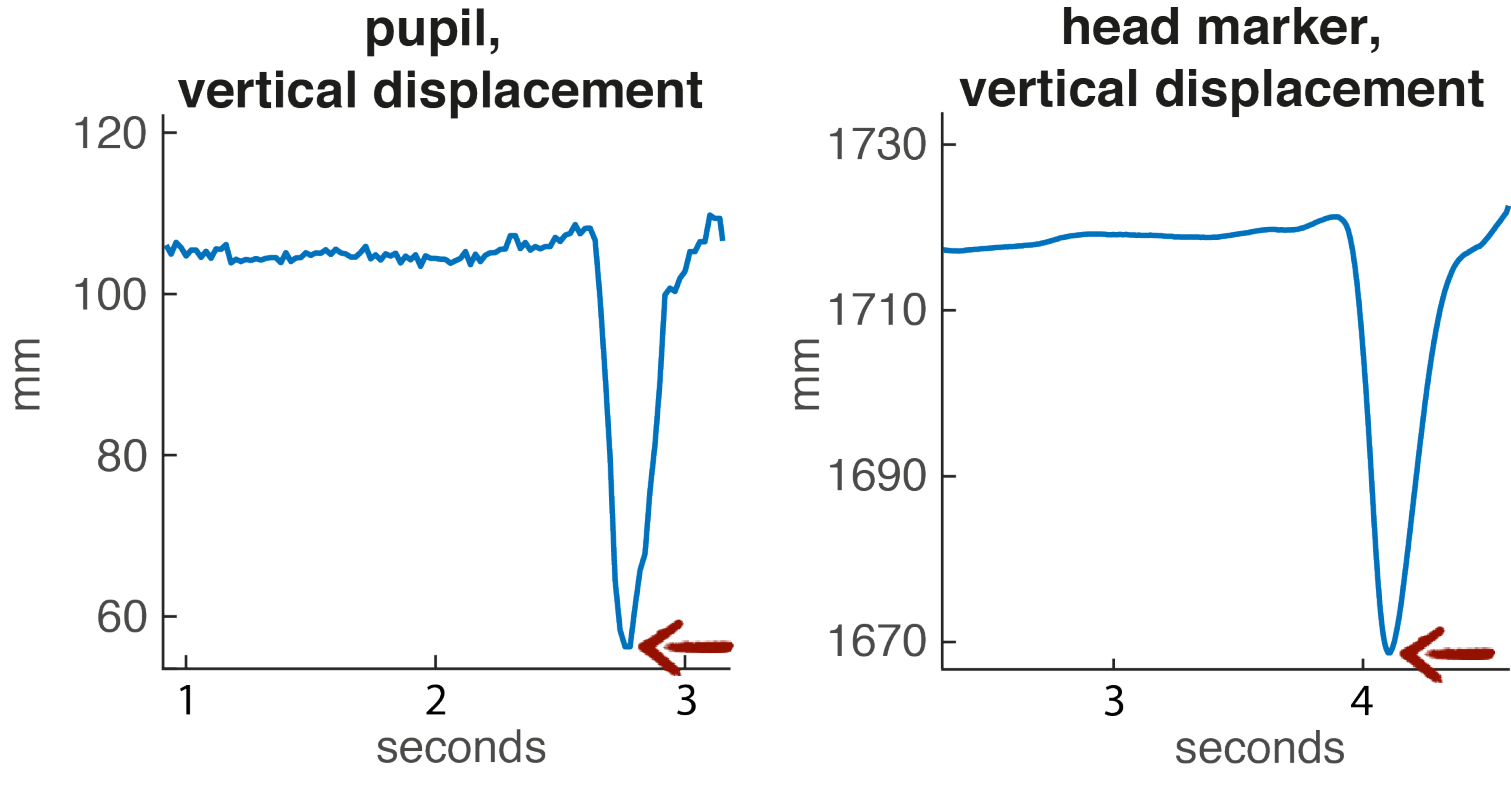
Vertical displacement of pupil and (front left) head marker during the nod (indicated by the red arrow).

In order to synchronize the eye tracker and motion capture recordings, the participants were instructed to look straight with upright body posture fixing a point in space (e.g., a target on the wall, in our case the head of another person standing in front of a wall opposite the participant), and then nod (i.e., move the chin towards the chest, change direction when being about half way between straight position and chest) very quickly at the beginning of each recording, keeping the eyes open and fixating the target while nodding (see mocap trace illustration in Fig. 2). The nod should be performed as one movement (i.e., not stopping in the lowest point of the motion, but just changing directions and moving upwards immediately). The nod resulted in a sharp vertical displacement of both the pupil data and the mocap data of the head markers (see Fig. 3) at the same time. The time point of maximal displacement is used to align mocap and eye tracker data afterwards. To simplify the procedure, the mocap recording was always started first, followed by the recording of the eye tracker, with about a second delay. The start of each recording was announced to the participants. Thus, sufficient time was given for the participants to adjust to the target before and/or right at the beginning of the recording.

### Analysis

The following workflow describes our approach to computationally synchronize eye tracker and mocap data. Several steps are required to prepare the data, so that automatic synching is possible.

The first step for the pupil data was performed in the Ergoneers recording software D-Lab, whereas all the remaining steps were performed in Matlab using the MoCap Toolbox ( 5 ), a Matlab toolbox for analyzing and visualizing motion capture data, and other Matlab functions. The first step in D-Lab included a pupil detection check to ensure that the pupil was successfully recognized during the nod. For those participants whose pupil moved out of the automatic tracking range when at the maximum of the nod, it was manually added using the “Pupil adjustment” function in D-Lab. Afterwards, the numerical data were exported into a text file.

After labeling the mocap markers in the respective recording software Qualisys Track Manager (QTM) and exporting the labeled data into a text file, both pupil and mocap data were imported into Matlab using the MoCap Toolbox. In order to process the eye tracker data, we wrote an extension that would read in the numerical output from the eye tracker software and parse it into a MoCap Toolbox compatible data structure.

**Figure 4.**
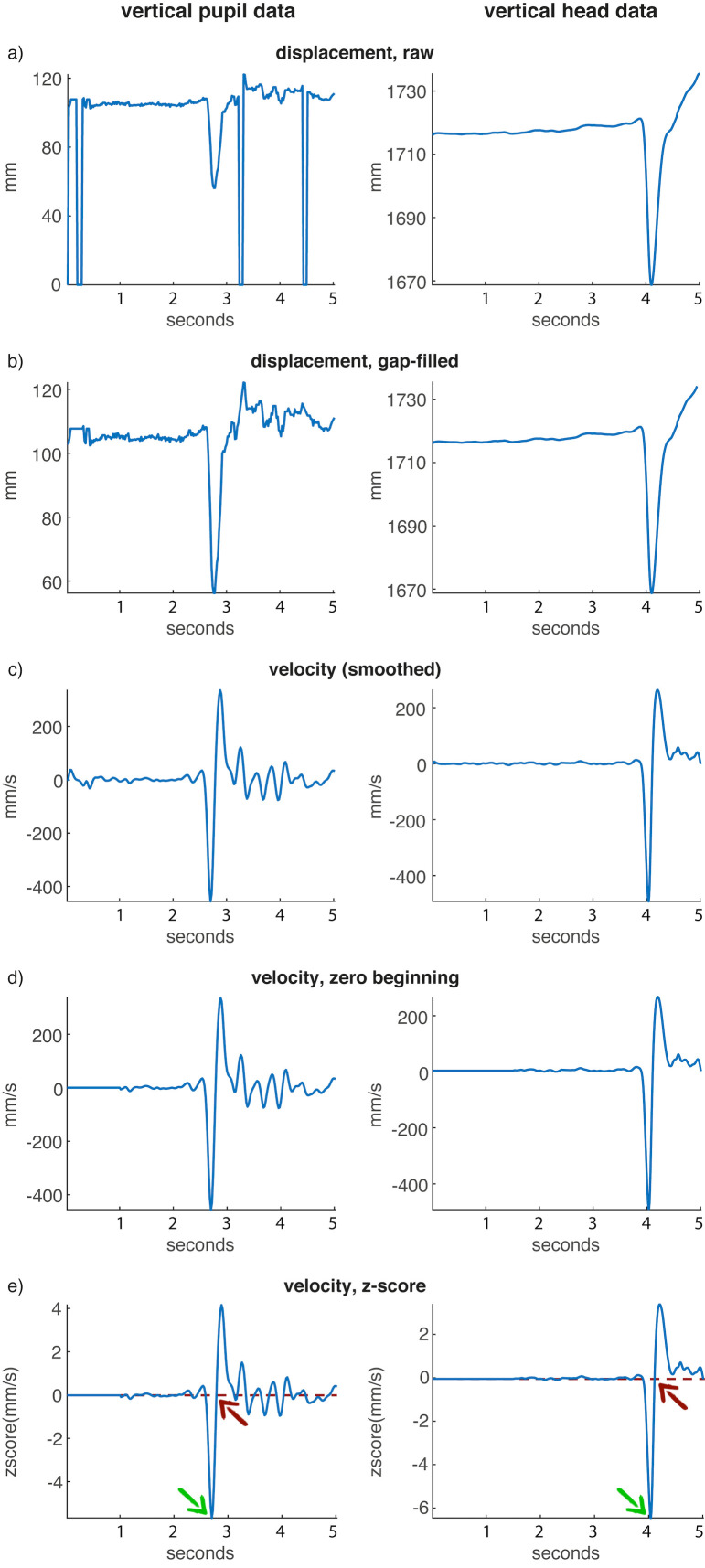
Workflow of the synchronization procedure. a) vertical displacement of pupil and left front head marker; b) linearly gap-filled data; c) vertical velocity data; d) first 1.5 sec of mocap and first sec of pupil data set to 0; e) z-scored data plus indicating peak-picking of velocity minima of the nod (green arrows) and subsequent zero-crossing (red arrows) used to align eye and mocap data.

For this analysis, the vertical displacement of the pupil data and the vertical displacement of the left front head marker was used (Fig. 4a). In the first step, the pupil data was linearly gap-filled to remove possible blinks happening before and after the nod (see Fig. 4b). This was done to make the data smoother (i.e., more continuous) for further computation. Gaps of blinks were short (max. 10 frames) and since these actual data were not relevant for the computation, the gaps could be filled irrespective of their length without necessitating the use of a threshold. The mocap data were gap-filled just in case, although it was rather unlikely that the head marker was occluded in the beginning of a recording.

Next, the instantaneous velocity was calculated for both eye and mocap data using numerical differentiation and a Butterworth smoothing filter (second-order zerophase digital filter). This centered the data around 0, removing potential differences in the height of the participants as well as compensating for movement drifts in the beginning (in case the participant was not yet looking at the target), while also resulting in a sharper, more focused minimum of the curve (representing the nod) compared to the position data (see. Fig. 4c).

In order to remove artefacts in the beginning of the recording (i.e., the participant was not focusing the target yet), the first second of the pupil data and the first 1.5 seconds of the mocap data were set to 0 (see Fig. 4d). This was possible to be done hazard-free, due to starting the recording devices consecutively at a relatively low pace (mocap was started first, thus the slightly longer time).

Subsequently, both data streams were z-scored (a way to standardize scores to have an overall mean of 0 and a standard deviation of 1) to adjust the scaling of the values, since some participants would nod faster than others (see Fig. 4e).

Following this, the first local minimum of each data stream was computationally determined by using a (selfimplemented) peak-picking algorithm with a threshold of -2 (see green arrows in Fig. 4e). This value was found suitable as a threshold in the given data set.

The maximal dislocation of position data (i.e., at the point of change in direction from moving the head downwards to moving the head upwards again) results in a velocity value of zero, so the zero-crossing of the velocity curve following the first local maximum was determined (see red arrows in Fig. 4e) and taken as the synchronization point. Since the velocity value would never be exactly zero, the frame before the zero-crossing was used as the synchronization point.

**Figure 5.**
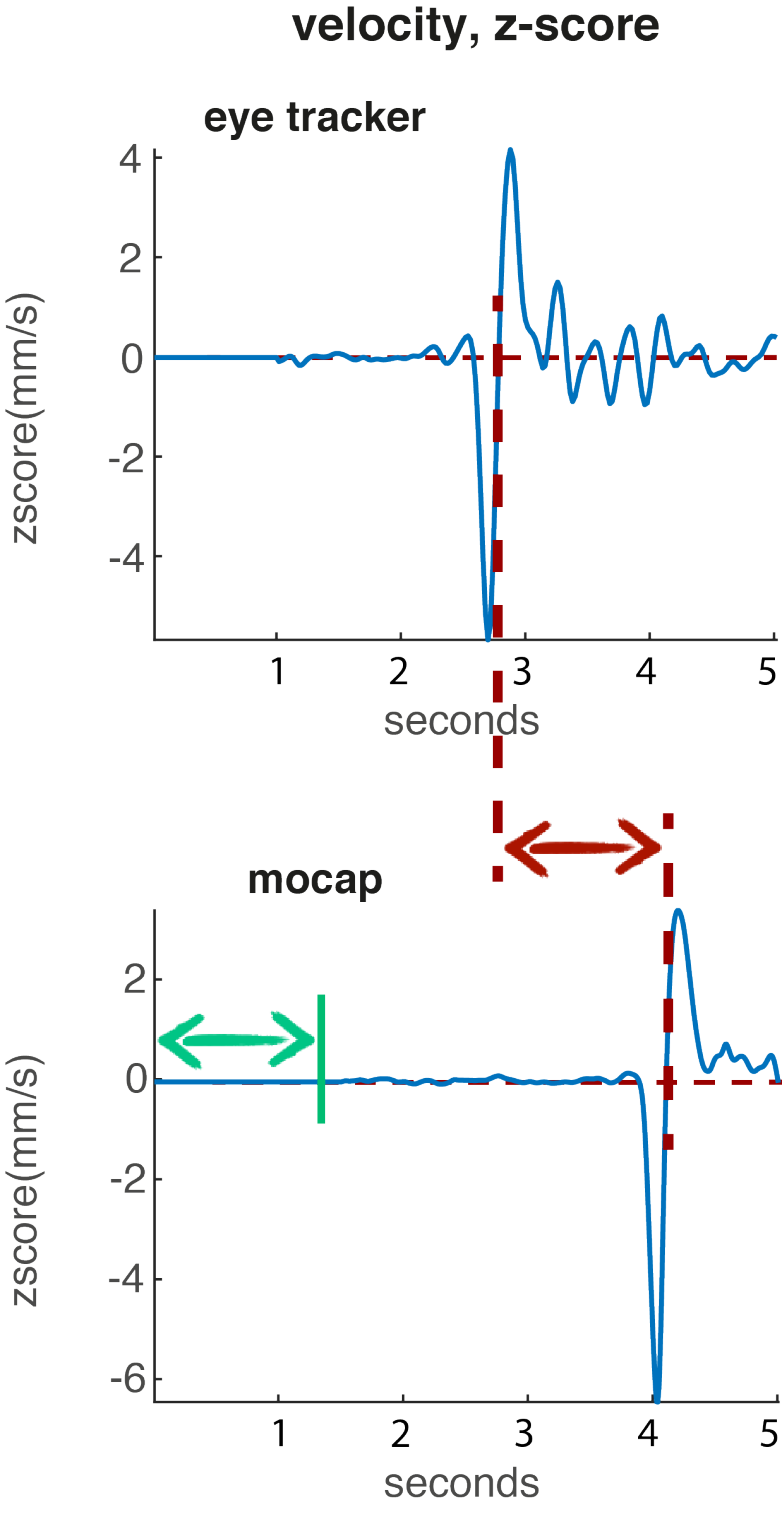
Illustration of the trimming. The dashed red lines indicate the sync points for each data stream. The red arrow indicates the temporal difference between both sync points. The green arrow (same size as the red arrow) indicates the part to be trimmed from the beginning of the mocap data.

In the last step, the (temporal) difference between the occurrences of both zero-crossings was calculated and subsequently used to trim the beginning of the mocap data, so that the nod would be aligned in both data streams and the data therefore synchronized (see Fig. 5).

### Results and evaluation

**Figure 6.**
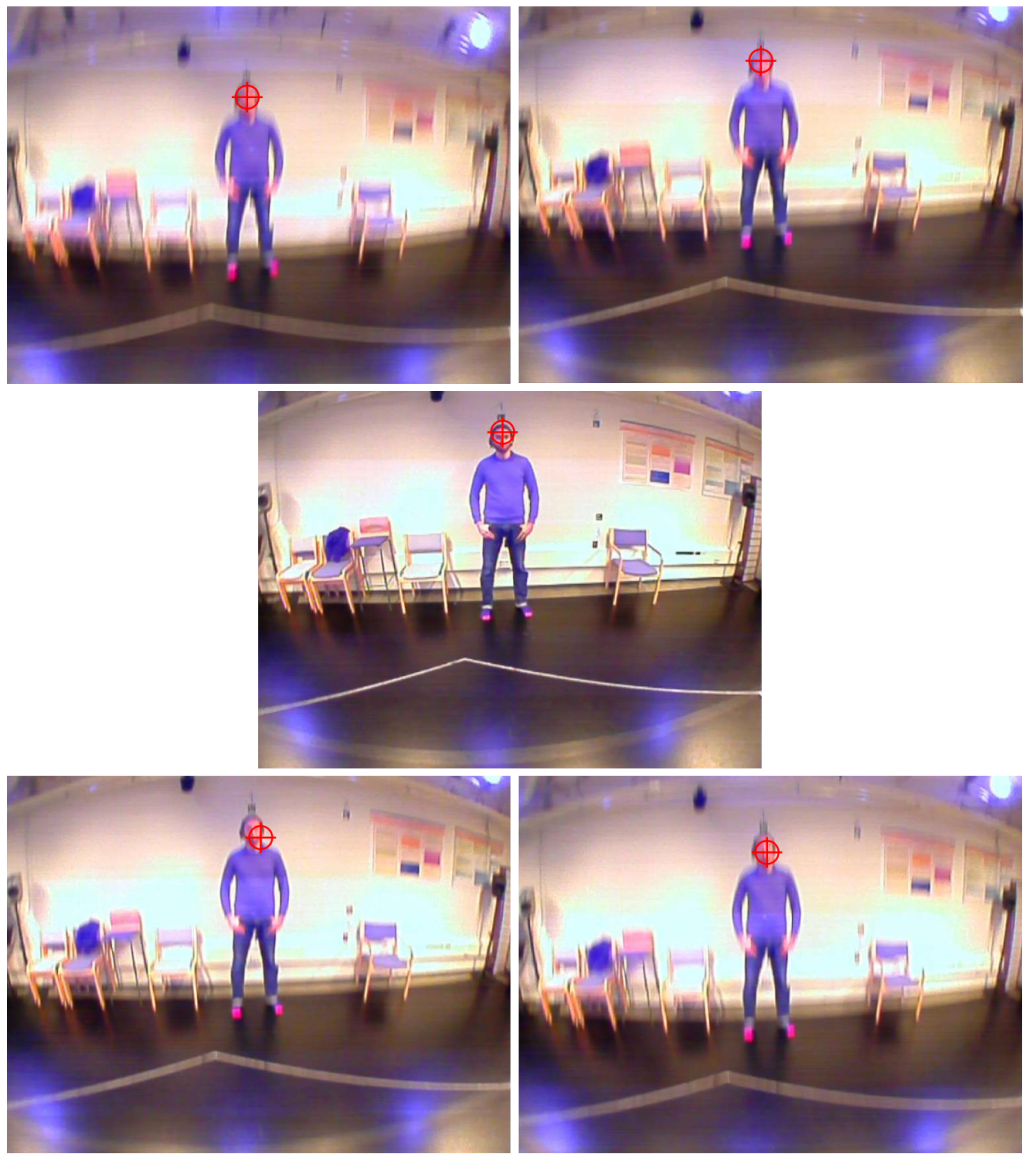
Field camera sequence of the head nod. During the nod, the head (i.e., field camera) moves downwards until the point of most downward displacement (middle picture) while fixing the target (the face of the other personin this case).The corresponding time stamp of the picture frame in the middle constitutes the “ground truth data”.

In order to test whether the computational extraction could locate the synch point correctly, “ground truth data” from both the mocap and the eye tracker were assessed for comparison purposes. The motion capture “ground truth data” were assessed within QTM by determining the minimal point of the vertical dislocation of the head during the nod in each recording by plotting the time series of the left front head marker. For the eye tracker data, this was done in D-Lab. The frame that displayed the most downwards displacement of the field camera was taken as the reference for the maximal vertical dislocation of the eye during the nod (for an example see Figure 6). For this, the play-back / time line was manually reset, so that the recording would start from 0:00 (this is due to D-Lab not using a global time code to record the different cameras, so starting times vary between eye and field camera).

Subsequently, the sync points of the computational extraction were subtracted from the “ground truth data” in order to determine the accuracy of the computational solution. The data, as well as their respective differences from two of the six participants, are presented in Table 1.

**Table 1.** “Ground truth” sync points (“QTM” and “D-Lab”) and computationally derived sync points (“Matlab”) as well as their respective difference (“Diff”). All values are in seconds.

	Mocap data			Pupil data		
Trial	QTM	Matlab	Diff	D-Lab	Matlab	Diff
1	4.7500	4.7500	0	3.48	3.46	-0.02
2	4.9000	4.9000	0	3.00	3.02	0.02
3	4.7830	4.7830	0	3.16	3.14	-0.02
4	4.7500	4.7583	0.0083	3.50	3.50	0
5	3.8830	3.8830	0	2.70	2.70	0
6	4.1083	4.1083	0	2.76	2.76	0.02
7	4.6916	4.6916	0	2.88	2.88	0
8	3.7500	3.7500	0	2.78	2.78	0
9	3.5916	3.5916	0	2.54	2.52	-0.02
10	4.5916	4.5916	0	3.02	3.02	0

For the mocap data, the “ground truth data” equaled the computationally-derived sync points in all trials but one, in which a difference of one frame (8.3 ms) was found. For the pupil data, the “ground truth data” conformed to the computationally derived sync points in five trials, whereas there was a difference of one frame (20 ms) in the other five. In three of these five trials, the difference was negative, meaning the automatic sync solution located the peak of the nod one frame before the “ground truth data”, whereas, in the other two trials, the automatic solution located the peak nod after the “ground truth data”. This suggests that the differences were rather due to rounding errors in the calculation than a trend that one measure would have been consistently behind the other.

### Discussion

The results of the comparison between “ground truth data” and computational synchronization show that the computational solution is able to correctly and accurately identify the nod. However, several issues arose that led to refinements of the approach. Only the data of two out of six participants could be synced this way. The reason for the two other participants failing was mainly that they blinked during the nod instead of keeping the eyes open. A technical weakness in our approach was that we only aligned the recordings in the beginning, leaving it unknown whether any drift or jitter would occur between the two data streams that would result in inaccurate synchronization. Furthermore, the sample was relatively small, so more data were needed to test and validate the method. Thus, several improvements to the approach were made, which are outlined in the following sections.

## Appraisal phase – Methods

This section will outline the second data collection used to test and validate the synchronization approach, alongside the changes to the method to improve the approach.

We again chose to collect data within an actual experiment setting. Ten participants signed four different sentences to another person standing opposite to them, resulting in four recordings per person and 40 recordings all together.

### Equipment

Data were recorded with the same eight-camera Oqus 5+ motion capture system, however this time tracking at a rate of 200 Hz. This was done to increase the temporal accuracy and to reduce rounding errors by matching the sampling frequency of the eye tracker in an integer relationship. The same Ergoneers head mounted eye tracker at a sampling rate of 50 Hz was used.

### Procedure

Two features were added to the procedure. In order to familiarize the participants with the environment and the nodding task, several practice nods were included at the beginning of the recording procedure, thus ensuring that the participant would understand the task and its requirements and perform it correctly, in particular keeping the eyes open during the nod. Furthermore, a nod in the end was added to test the accuracy of the synchronization and whether there was any drift in the system (due to technical challenges to use time coding with the eye tracker). Since the recordings of the four sentences were rather short (recordings lasting 10 to 15 seconds), we recorded five longer conversations of about 1 minute each with a subset of five of the ten participants. As was done before, the mocap recording was started before the eye tracker.

### Subjective experience of participants

In order to investigate the subjective experience of the nodding procedure, participants were asked to fill in a short questionnaire after the data collection. Questions related to how easy it was to keep the eyes open during the nodding, how comfortable participants felt during the nod, how clear it was when to produce the nod, and how much the participants felt that the nod disturbed the performance of the main task of the data collection. The items were rated on 7-step scales ranging from “not at all” to “very much”.

### Analysis

**Figure 7.**
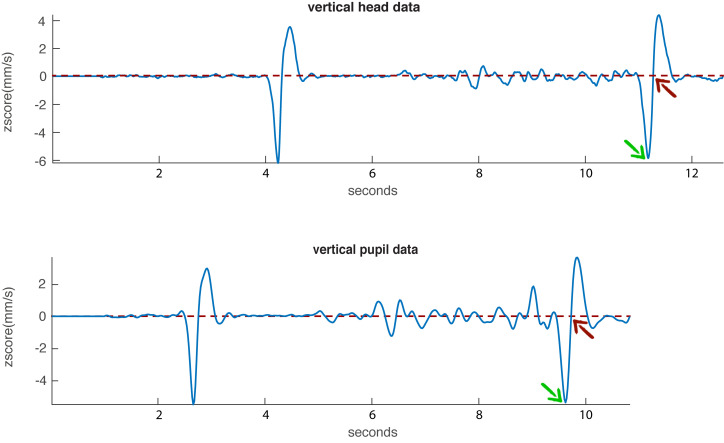
Illustration of the end nod with the z-scored velocity data. The green arrow indicates the local minima exceeding the threshold of -2 corresponding to the end nod. The red arrow indicates the subsequent zero-crossing that was used as sync point.

The sync points were computed in the same way as described above. In order to locate the nods in the end of the recording, the algorithm was used in the reversed way, so starting the peak detection from the end of the recording. Whenever the first local minima exceeding the threshold of -2 was reached, the algorithm would revert to the previous zero-crossing and choose the frame before the zerocrossing as the sync point. However, the recording was not trimmed, so the sync point could be expressed relative to the beginning of the recording. An exemplification is shown in Figure 7.

Moreover, the “ground truth data” of mocap and eye tracker were gathered in the same way as previously. However, it was now performed for both the nod in the beginning and the nod in the end.

## Results and evaluation

We will first present the results regarding the alignment of computationally extracted sync points and the manually acquired “ground truth data” to evaluate the accuracy of the sync point extraction. Table 2 displays the differences between the temporal locations from the computational synchronization approach and the manual “ground truth data” of both mocap and eye tracker for each of the 40 recordings. The differences are given in frames.

**Table 2.** Differences in frames between the computational sync points and the “ground truth data” of both mocap and eye tracker for all 40 recordings. One mocap frame equals 5 ms, while one eye tracker frame equals 20 ms.

	mocap		eye tracker	
	start nod	end nod	start nod	end nod
P1	0 | 0 | 0 | 0	0 | 0 | 0 | 0	0 | 0 | 0 | 0	0 | 0 | 0 | 0
P2	0 | 1 | 0 | 0	0 | 0 | 0 | -1	1 | 0 | 0 | 0	0 | 0 | 0 | 0
P3	0 | 0 | 1 | 0	0 | 0 | 0 | 0	0 | 0 | 0 | 0	1 | 0 | 0 | 0
P4	0 | 0 | 0 | 0	1 | 0 | 0 | 0	0 | 0 | 0 | 0	0 | 0 | 0 | 0
P5	0 | 0 | 0 | 0	0 | 0 | 0 | 0	0 | 0 | 0 | 0	1 | 0 | 0 | 0
P6	0 | 0 | 0 | 0	0 | 1 | 0 | 0	0 | 0 | 0 | 0	0 | 0 | 0 | 0
P7	0 | 0 | 0 | 0	0 | 0 | 0 | 0	0 | 0 | 0 | 0	0 | 1 | 0 | 0
P8	1 | 0 | 0 | 0	0 | 0 | 0 | 0	0 | 0 | 1 | 0	0 | 0 | 0 | 1
P9	0 | 0 | 0 | 1	0 | 0 | 0 | 0	0 | 0 | 0 | 0	0 | 0 | 0 | 0
P10	0 | 0 | 0 | 1	0 | 0 | 0 | 0	0 | 1 | 0 | 1	0 | 0 | 1 | 0

For the mocap data, the “ground truth data” equaled the computationally derived sync points in all but five trials for the nod in the beginning and three trials for the nod in the end. In all cases, the difference was one frame (5 ms). For the pupil data, the “ground truth data” conformed with the computationally derived sync points in all but four trials for the nod at the beginning and five trials for the nod at the end. Each difference was also one frame (20 ms) in these instances. In all cases but one (P2, end nod mocap), the sync point was one frame after the “ground truth data”.

In order to further evaluate the accuracy of the synchronization solution regarding synchronization over time of both systems (i.e., drift), the durations in between the nod at the beginning and the end for the mocap system and the eye tracker were compared per trial. The results of the short recordings are shown in Table 3, while the five longer recordings are presented in Table 4.

**Table 3.** Differences of recording durations in seconds of mocap system and eye tracker between beginning and end nod per trial and average per participant.

	Duration differences per trial in seconds				
	T1	T2	T3	T4	Mean
P1	0.010	0	0.005	0.005	0.0050
P2	0	0.005	0	-0.030	0.0088
P3	0.005	-0.010	0	0.015	0.0075
P4	-0.020	0	-0.010	-0.020	0.0125
P5	0.005	-0.005	-0.005	0	0.0038
P6	0	0	0	0	0
P7	-0.005	0.010	0.020	0.010	0.0112
P8	-0.010	0.005	-0.005	0.005	0.0063
P9	0.005	0	0	-0.005	0.0025
P10	-0.010	-0.005	0	0	0.0037

**Table 4.** Differences of recording durations in seconds of mocap and eye tracker for long recordings.

	Durations in seconds		
	Mocap	Eye	Difference
R1	42.700	42.700	0
R2	43.010	43.000	-0.010
R3	48.895	48.880	-0.015
R4	83.895	83.860	-0.035
R5	65.815	65.800	-0.015

For the short recordings, the duration differences are on average below the sampling frequency of the eye tracker (50Hz, 20ms); in eight out of ten cases, the average difference is below half the eye tracker sampling frequency. In 35 of the 40 recordings, the difference is smaller than or half of the eye tracker sampling frequency. In 14 cases of the short recordings, the eye tracker and the mocap recordings were of exactly the same length, whereas in 13, the eye tracker recording was shorter than the mocap, and in the remaining 13, the eye tracker recording was longer than the mocap.

In the five longer recordings, the differences ranged from 0 to 0.035 ms, with four of the five recordings being below the eye tracker frequency. For all long recordings, the eye tracker recordings were shorter compared to the mocap system.

### Subjective experiences

Participants were asked to rate four questions regarding their experiences about the nod after the data collection on a 7-point scale. The detailed overview of the ratings is found in Table 5.

**Table 5.** Rating results of participants’ subjective experiences. A 7-step scale (1=not at all – 7=very much, reversed for last question) was used.

	Mean	Standard deviation
How easy was it to keep the eyes open during the nods?	5.6	1.65
How comfortable did you feel during the nod?	5.0	1.89
How clear was it when to produce the nod?	6.4	0.97
How disturbing was it to perform the nod?	2.3	1.83

Participants were overall positive about the task. They found it easy to keep the eyes open and were overall rather comfortable with the task. It was very clear when to produce the nod. Furthermore, the nod was not perceived as disturbing.

## Discussion

In this paper, we described the development of a computational approach to automatically synchronize recordings of a motion capture system and an eye tracker. The aim of the paper was to present a solution that is reliable and does not depend on a ready-made plug-in by the manufacturer, but is instead device-free and intrinsic to the recording of the data.

The measured accuracy of the motion capture data is very high; 90% of the (nine out of ten) pilot recordings at a frame rate of 120 Hz, and 90% (72 out of 80) of the second data collection at 200 Hz could be optimally aligned between the “ground truth data” and the computational solution, while the remaining ones showed one frame difference. The difference of one frame (8.3 ms at 120 Hz and 5 ms at 200 Hz) could be due to the smoothing of the data after the time derivation or due to rounding during the calculation. Small inconsistencies could also have emerged from a slower speed or a smoother movement during the nod. However, the time difference is so small that it can be considered negligible.

The accuracy of the eye tracker data was less than the mocap data in the pilot recordings, though it increased during the second data collection. In the pilot, five out of the ten recordings (50%) could be optimally aligned, whereas the remaining five differed in one frame each. In the second data collection, 71 out of 80 sync points equaled the “ground truth data” (88.75%). These values suggest that the procedure can be regarded reliable and accurate for the required purpose of time-critically synchronizing both systems. Our analysis showed a maximum difference of one frame in each system, suggesting a maximum difference (“worst case scenario”) of 25 ms between mocap and eye tracker, while the actual differences were mostly much smaller. These values should be sufficient for most research questions related to eye movement, unless very fast saccades and microsaccades are of interest ( 16, 40 ). However, if higher temporal accuracy of the eye movements is needed, the sampling frequency of the eye tracker should be (much) higher than 50 Hz.

We increased the sampling frequency of the mocap system from 120 Hz to 200 Hz to match the eye tracker sampling frequency in an integer relationship. Despite recording more data points per time, this did not increase the accuracy of locating the sync points (peaks of the nods), as we received the same percentage of correctly located synch points. However, it might have still reduced rounding errors when combining the data with the eye tracker and thus increased data accuracy when trimming the data, due to less noisy rounding and interpolation between the two systems.

The less accurate synchronization result for the pupil data, especially in the pilot recordings could be related to the “ground truth data” being based on a video signal and not a time series data representation, like the mocap data. Local minima of a curve might be more clearly detectable than the change in frames of the eye tracker video data, thus that kind of “ground truth data” could be slightly less reliable. Issues in pupil detection (i.e., when the pupil was adjusted manually) could also have influenced the accuracy. Manual adjustment might have caused less accurate precision or larger differences between continuous frames than automatic tracking, thus the resulting velocity curve could have contained more noise. Furthermore, slight inconsistencies could have emerged due to different starting points of the field and the eye camera. D-Lab does not use a global time clock for its recordings, but records the devices “as they are detected”, so there has been a variable delay (ranging from 14 ms to 85 ms) between the start of both cameras.

When trimming the data and comparing the resulting lengths of both recordings, the recordings were very similar in length. In most cases, the differences were below the sampling frequency of the eye tracker (often even half the sampling frequency), so the accuracy should be sufficient for most applications as mentioned above. The small differences in the lengths of the recordings could be related to rounding errors when deriving the sync points, or suggest that there is a bit of drift in the alignment of the two data streams. Since the differences in the shorter recordings are both positive and negative (i.e., for some recordings, the eye tracker recording is shorter, whereas in other cases the mocap recording is shorter), these might be rather due to rounding errors, whereas in the long recordings, the eye tracker recordings were all shorter than the mocap, suggesting a trend that the eye tracker was “faster”. For a more extensive investigation of the existing drift as well as possible jitter, appropriate hardware is required that would synchronize the recordings using genlocking on a frame-to-frame basis.

Moreover, our longer recordings of about one minute were still relatively short. In order to further investigate drift and jitter between the motion capture system and eye tracker, longer recordings (e.g., about 10 minutes) should be made. However, since recordings in our studies are usually not longer than one to two minutes, we refrained from making longer recordings at this stage.

In the pilot data collection, only two out of six participants could be reliably synchronized using this approach. The other four were found difficult due to different reasons. In two cases, the eye tracker could not reliably track the participants’ pupils due to technical difficulties. In the other two cases, the participants were blinking at the moment of the nod. The closure time in these cases was in the middle of the nod, so it was impossible to manually adjust (or add/estimate) the pupil, after which the computational synchronization could still have been possible. In the second data collection, we provided the participants with more thorough and clear instructions, as well as asked them to perform practice nods prior to the recording to make them familiar with the procedure. This seemed to have clearly helped, as none of the participants blinked during the nod in the second data collection. This finding strongly indicates the importance of clear instruction for the participants, explaining the procedure to them, and ensuring they understand the underlying rationale.

The assessment of participants’ subjective experiences related to the nod showed that it was not perceived as disturbing or difficult to perform. It seemed to have been well integrated into the task and was clear when and how to produce it. It might have also helped participants to have a defined start and end of each recording and concentrate on the task. In order to even further prompt the participants to perform the nod, a metronome beat could be presented (in case of hearing participants), so that the participant could synchronize the nod for instance to the fifth beat.

In order to check that the nod was performed successfully, a real-time or close to real-time check could be included. If it was possible to, for instance, display the vertical displacement of the eye movement as a time-series directly during the recording, the success of the nod (especially whether or not a blink happened) could be checked immediately after it was performed.

The question whether more accurate results would have resulted from the synchronization plug-in provided by Qualisys or a sync box solution remains. The technical setup of the plug-in involving two wirelessly connected computers might point towards such connections potentially introducing lags. However, in order to answer this sufficiently, the set-up would have to be tested with both the plugin and the nod and the results compared afterwards.

We also considered other motion sequences for the synchronization in order to potentially improve our approach and make the synchronization easier. We piloted two different approaches, 1) several consecutive nods and 2) a passive application of force by someone else exerting a sudden strike to the participant’s head. However, our volunteers found both approaches more uncomfortable than the single nod. The consecutive nods felt very unnatural, and either the first or the last were less pronounced, making it difficult for the automatic detection to choose one. The sudden knock felt uncomfortable, since, despite knowing about it, it still felt somewhat unexpected. Additionally, volunteers involuntarily blinked during the nod, probably due to the sudden and unexpected exertion of force on them. It seems, therefore, that a single nod is the best approach for this method.

The approach described here will be integrated into the Mocap Toolbox to make it assessable for everyone. As of now it is available for free on the toolbox website (MoCap Toolbox website: https://www.jyu.fi/hytk/fi/laitokset/mutku/en/research/materials/mocaptoolbox) and will be integrated into the toolbox with the next release. The function set includes the function to read the Dikablis eye tracker data into Matlab, convert it into a Mocap Toolbox compatible data structure, and the function to automatically sync the eye tracker recording with the corresponding mocap recording. No further Matlab expertise than basic understanding of how to use the Mocap Toolbox nor any other external devices would be needed to apply this syncing method. Furthermore, since the Mocap Toolbox stores the eye tracker data in the same way as mocap data, the same functions and procedures can be used to analyze the eye tracker data.

The mocap toolbox function also offers the possibility to adapt the thresholds for detecting the nod for both mocap and pupil data. In our data sets, a threshold of -2 could reliably detect the nod in both (the z-scored) mocap and pupil data, though this might not be the case for other recordings. Thus, adjustable thresholds that can account for participants performing the nod at different speeds and spans make the function more flexible.

**Figure 8.**
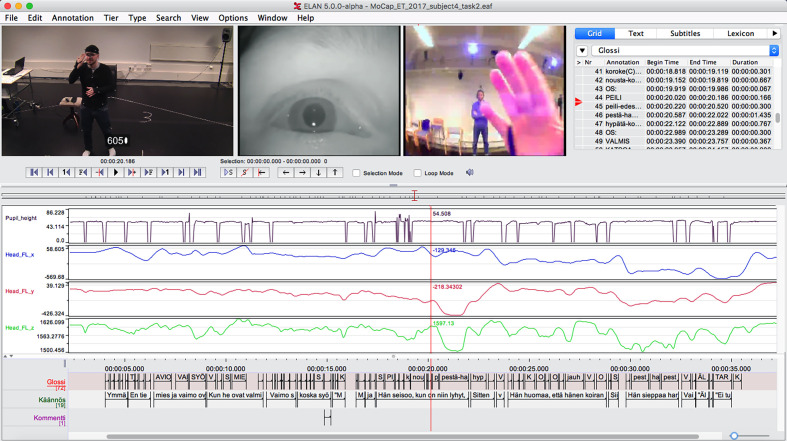
Screenshot of the ELAN multimedia annotation software. The upper part of the screen shows video data from the external video camera as well as from the pupil and field camera. The descriptors in the middle visualize the pupil height data (the upmost panel) and the three-dimensional marker location data derived from the front left head marker (the three bottom panels). The markings on the three tiers at the bottom of the screen are annotation cells time-aligned with the video data.

Furthermore, the nod was useful for manually synchronizing the different data streams used in the experiment. The motion capture, eye tracker, and regular video data could be accurately synchronized by using the nod as a reference when importing the data into the freely-available audio and video annotation and transcription software ELAN (see screenshot in Fig. 8), developed at the Max Planck Institute for Psycholinguistics, The Language Archive, Nijmegen, The Netherlands. Given the different sampling frequencies of all the systems, it would have been much more difficult to synchronize the recordings without the clear displacement that the nod provided.

## Conclusions

This paper presented a generic, device-free approach to accurately synchronize eye tracking and motion capture systems computationally. Since it is a behavior-based approach, it is expected to work with every motion capture and (mobile) eye tracking system. The method has so far only been tested with one motion capture system and one eye tracker, thus it should be tested with a wider range of systems in the future. Careful instruction of participants is crucial in this approach, so that they are aware of what they are supposed to do. Nevertheless, with participants performing in the desired manner, the approach offers an easy and device-free possibility to accurately synchronize both devices. This method can be especially useful in case plugin solutions are not available, are technically too demanding, or are too cost-intensive. Furthermore, when external devices, such as regular video cameras need to be synchronized as well, this method has shown to be beneficial.

### Ethics and Conflict of Interest

The authors declare that the contents of the article are in agreement with the ethics described in http://biblio.unibe.ch/portale/elibrary/BOP/jemr/ethics.html and that there is no conflict of interest regarding the publication of this paper.

### Acknowledgements

This study was supported by the Academy of Finland (projects 299067, 269089, and 304034). We wish to thank Emma Allingham for help with the eye tracker and Elsa Campbell for proofreading the manuscript.
